# Supplementary material for: Multi-source heterogeneous blockchain data quality assessment model for enterprise business activities
Source: PLoS One. 2024 Jun 14;19(6):e0304835. doi: 10.1371/journal.pone.0304835 (PMC11178169; doi:10.1371/journal.pone.0304835)
Supplement: Attachment — Submitted filename: Response to Reviewers.docx [file pone.0304835.s001.docx]

Responses to Reviewers’ Comments

## Dear Editor and Reviewers,

Thanks for your comments concerning our manuscript entitled “multi-source heterogeneous blockchain data quality assessment model for enterprise business activities". The comments are very valuable and helpful for revising and improving our manuscript. We have carefully revised the manuscript and responded all comments below. The changes will not affect the content and framework of the paper. The revised sentences are highlighted in yellow in the manuscript.

Best regards,

Baoyan Song

# Reviewer 1:

# 1- Experimental Details and Data. The manuscript lacks specific details on experimental setups, controls, replications, and sample sizes. To ensure the technical soundness of your research, it is essential to provide comprehensive information about the experiments conducted, including the datasets used, methodology for control, and the size of the samples involved.

## Thanks for the reviewer 's advice. We have added a separate subsection to the experimental section that describes the experimental setting and experimental dataset of this paper, as well as other models that were tested in comparison with the model proposed in this paper. The details are as follows.

***Experiments setting***

*The experimental hardware and software environment is shown in Table 1.*

*Table 1 Experimental platform sets*

| *Hardware* | *Bytes* |
| --- | --- |
| *CPU* | *Intel Core i5-7400* |
| *CPU running* | *3GHz* |
| *Memory* | *8GB* |
| *Operation system* | *Windows11* |
| *Programming environment* | *PyCharm 2020.3.3* |
| *Programming language* | *Python* |
| *Programming language version* | *3.7.3* |

*The experimental dataset is searched on search engines with the subject term of business activities and crawled official data, about 110,000 items, and then simple data cleaning is performed by filling in missing values, smoothing or removing outlier points and other operations to get about 100,000 items of data, and the details of the dataset are listed in Table 2. In this paper, simulation experiments are conducted in terms of model parameter taking and model evaluation efficiency respectively, and the model built in this paper (DQAM) is used to compare with other models, and the comparison models are AHP, DSMM, etc.*

*Table 2 Experimental data sets*

| *Entity Name* | *Bytes* |
| --- | --- |
| *name* | *16bytes* |
| *type* | *8 bytes* |
| *activityOntology* | *Variable length bytes* |
| *comment* | *Variable length bytes* |
| *informationSource* | *16 bytes* |
| *code* | *4 bytes* |
| *registrationDay* | *4 bytes* |
| *capital* | *16 bytes* |
| *address* | *32 bytes* |

# 2- Clarity in Methodology. The methodology section, especially in Section 3.1 regarding the CEKGRL model, is intricate and could benefit from additional clarity. A more detailed explanation of the entity triad representation, attention mechanism, and how the model addresses inconsistencies in entity information would enhance the reader's understanding.

## Thanks for the reviewer 's advice. We have reorganized the logic of Section 3.1 and added additional explanations related to the entity triad representation, the attention mechanism, and how the model resolves inconsistencies in entity information. The details are as follows.

The triple structure is a data model widely used for representing information, consisting of three components: subject, predicate, and object. It is extensively applied in fields such as knowledge graphs and the semantic web. The triple structure is highly flexible and can be employed to represent various types of information. Different combinations of subjects, predicates, and objects can capture rich semantic relationships, making it suitable for diverse knowledge representation. In summary, the advantages of the triple structure lie in its flexibility, clear semantic representation, scalability, and effective expression of relationships, making it a key technology in the fields of knowledge representation and the semantic web.

# 3- Contextual Information and Ambiguity. While the introduction of contextual information for similarity calculation is interesting, further details are needed to understand how this addresses the "one-to-many" mapping problem and ambiguity in entity name designation. A clearer explanation of how the contextual information association graph model contributes to resolving these issues would be beneficial.

## Thanks for the reviewer 's advice. We have added a description of contextual information and ambiguity in Section 3.2. We explain specifically how contextual information solves the "one-to-many" mapping problem and the ambiguity of entity name designation.

In traditional blockchain enterprise business activities information, there may be a situation where one entity has similarity with multiple different entities, which is called a "one to many" mapping problem. By introducing contextual information, a contextual information association graph model can be established to more accurately capture the relationships between entities.

# 4- Source Credibility Model. The credibility characterization of information sources in Section 3.4 requires more explanation, particularly regarding the calculation of the click-through rate and how it directly correlates with credibility. Please provide more insights into how this model aligns with established credibility standards.

## Thanks for the reviewer 's advice. We have added a description related to the information source credibility model in section 3.4, explaining the process of calculating the click-through rate and how it relates to credibility. We also explain the way in which the model is aligned with existing credibility standards. The details are presented below.

The click through rate is considered an important basis for measuring the credibility of information sources. Firstly, the click through rate reflects the level of interest and recognition of users towards the content of the information source. Users are more likely to click on information they consider useful, authentic, and trustworthy. Therefore, information sources with high click through rates often imply user trust in the source, thus becoming one of the indicators for evaluating credibility. Secondly, click through rate is a direct feedback of user behavior. The behavior of users clicking on a certain information source indicates that they are interested in the content of that source and believe that the content is valuable to them. This positive user behavior feedback is seen as an affirmation of the credibility of the information source.

# 5- Comment-Based Information Credibility. The methodology for comment-based information credibility characterization (Section 3.4) could be further detailed. Consider providing examples or case studies to illustrate how explicit and implicit comments are classified and contribute to the overall credibility assessment.

## Thanks for the reviewer 's advice. We have added a related description of comment-based information credibility in Section 3.4, with example explanations that illustrate how explicit and implicit comments can be categorized and how they can contribute to the overall credibility assessment. The details are as follows.

Explicit and implicit comments have different contributions to the evaluation of information credibility based on comments. These two types of comments reveal different attitudes and perspectives of users when facing information. By comprehensively analyzing them, the credibility of information can be more comprehensively evaluated. Explicit comments usually contain clear attitudes, opinions, or feedback from users. This direct expression helps to understand the user's true view of the information, thereby providing direct clues about the credibility of the information. And Explicit comments often contain more facts, data, or specific in-formation. This clear statement makes it easier to evaluate the accuracy of information and helps confirm whether the information is based on verifiable content. Implicit comments may involve emotional vocabulary, emotional color, or suggestive language, revealing the user's emotional feedback. This is of great significance for evaluating the user experience and impact of information. Implicit comments can provide additional con-text or information for explicit comments. Sometimes users may not express themselves clearly in their words, but through the tone and emotions in implicit comments, they can gain a deeper understanding of their views. These implicit viewpoints help to gain a more comprehensive understanding of the user's overall perception of information, providing more dimensions for credibility assessment.

# 6- Content-Based Credibility Characterization. The content-based credibility characterization assumes a direct relationship between the number of feature words and professionalism/credibility. It is important to discuss potential limitations of this assumption and consider other factors that may influence credibility.

## Thanks for the reviewer 's advice. We have added a relevant description of the content credibility characteristics in the last paragraph of Section 3.4. The details are as follows.

Some situation may affect the method like different fields may have different professional standards and vocabulary usage habits. Some fields may place more emphasis on the use of professional terminology, while others may place more emphasis on common language. Therefore, the relationship between the number of feature words and professionalism may vary between different fields. Sometimes, the number of feature words does not always reflect the depth and detail level of information. Some content may require more professional terminology and detailed explanations, which may not necessarily lead to an increase in the number of feature words. Therefore, quantity cannot fully represent the professionalism and credibility of the text. But in most cases, these issues will not have a significant impact on the conclusions of the model output, so no additional discussion is needed.

# 7- Conclusion and Implications. The conclusion should summarize the key findings and their implications. Discuss the potential limitations of the proposed method and suggest avenues for future research to strengthen the manuscript's overall impact.

## Thanks for the reviewer 's advice. We have reorganized the conclusion section and added a description related to the limitations of the paper's methodology and future perspectives as follows.

This paper has conducted in-depth research on the blockchain data quality evaluation model, proposed corresponding solutions for the shortcomings of current evaluation methods, and achieved some meaningful research results. However, the application of blockchain technology involves other fields besides the financial field. For its different application scenarios, the following issues related to this study need further exploration and research:

(1) The blockchain data quality evaluation model in this paper is aimed at the business activities data in-formation of enterprises in the financial field, while other fields are also producing a lot of data information at all times. Therefore, applying the evaluation model proposed in this paper to data in other fields is the next research direction.

(2) The model proposed in this paper is based on the established blockchain, but in reality, the information in the blockchain is sometimes stored dynamically, and may be linked at any time. Therefore, when new block information is generated in the blockchain, how to evaluate its quality in real time is worth studying.

# Reviewer 2:

# 1- The abstract needs refinement. Clearly articulate the role of the proposed study and highlight the actual contributions. This will provide readers with a better understanding of the paper's significance.

## Thanks for the reviewer 's advice. We have refined the abstract by adding a relevant description of the problem and significance of the paper to be studied as follows.

Blockchain-based applications are becoming more and more widespread in business operations. In view of the shortcomings of existing enterprise blockchain evaluation methods, this paper proposes a multi-source heterogeneous blockchain data quality evaluation model for enterprise business activities, so as to achieve efficient evaluation of business activity information consistency, credibility and value. This paper proposes a multi-source heterogeneous blockchain data quality assessment method for enterprise business activities, aiming at the problems that most of the data in enterprise business activities come from different data sources, information representation is inconsistent, information ambiguity between the same block chain is serious, and it is difficult to evaluate the consistency, credibility and value of information. The method firstly proposes an entity information representation method based on the Representation learning for fusing entity category information (CEKGRL) model, which introduces the triad structure of related entities in blockchain, then associates them with enterprise business activity categories, and carries out similarity calculation through contextual information to achieve blockchain information consistency assessment. After that, a trustworthiness characterization method is proposed based on information sources, information comments, and information contents, to obtain the trustworthiness assessment of the business. Finally, based on the information trustworthiness characterization, a value assessment method is introduced to assess the total value of business activity information in the blockchain, and a blockchain quality assessment model is constructed. The experimental results show that the proposed model has great advantages over existing methods in assessing inter-block consistency, intra-block activity information trustworthiness and the value of blockchain.

# 2- Expand the introduction to provide more context and background information. A more detailed introduction will better set the stage for the problem you are addressing, helping readers grasp the significance of your research.

## Thanks for the reviewer 's advice. We have added a description in the third paragraph of the introduction of the Section 1 to illustrate some of the problems of traditional enterprise blockchain, to better introduce the background information of the problem to be researched in this paper, and to highlight the significance of the research in this paper. The details are as follows.

In the enterprise blockchain data layer, each distributed node encapsulates the business activity information received over a period of time into a data block and links it to the longest blockchain in the current blockchain network, forming a new block. At the same time, with the increasing channels for obtaining information on enterprise business activities in blockchain, the amount of data is increasing. This information may originate from different institutions and fields, and there may be certain differences in the representation of information. Its credibility and information value cannot be measured, making it difficult to determine the overall quality of blockchain.

# 3- Include a section that compares your work with previous studies and techniques. This comparison can provide additional support for the validity and novelty of your research. Highlight similarities, differences, and advancements over prior work.

## Thanks for the reviewer 's advice. We have added a description in the last paragraph of Section 2 explaining the results achieved by building the new model as follows.

In summary, this paper proposes a multi-source heterogeneous blockchain quality assessment model for enterprise business activities by addressing the shortcomings of existing blockchain enterprise business activity information assessment methods and considering the consistency, credibility and value of data [21] and improve the efficiency of blockchain consistency assessment and considered the credibility of content, while achieving good results in situations with low information uncertainty.

# 4- Suggest a more in-depth analysis of security and privacy concerns associated with multi-source blockchain data. Encourage the inclusion of case studies or practical examples to demonstrate the model's application in real business scenarios and also Propose collaborations with blockchain enterprises for real-world testing and validation of the model.

## Thanks for the reviewer 's advice. The scheme proposed in this paper has been applied to projects such as "the first two funding" and in the actual business scenarios of Shenyang Fanshi Science and Technology Company Limited. Examples are added in Section 3.7, and the analysis of experimental results using real enterprise datasets is added in the experimental part. At this stage, the scheme of this paper focuses on the quality assessment of blockchain, and further in-depth analysis of security and privacy issues will be carried out subsequently. The details are as follows.

3.7 Instance

This section mainly evaluates the quality of our existing enterprise blockchain business activities, and obtains the final blockchain data quality evaluation result through comprehensive consistency, accuracy, and value calculation results. We will use effective data obtained from local enterprises to apply in this section.

Firstly, based on the credibility of the information source, it is mainly measured comprehensively by the credibility of the information source, information page, and publisher. For the credibility of the information source, its click through rate is calculated as click through rate=click through rate/exposure rate (as used in the example in this article); For the credibility of information pages, it is mainly measured by the accessibility of the page where the information is located and whether it is available after accessibility, while the credibility of publishers is judged by users through certain knowledge accumulation. Based on the calculation results of the above three factors, the credibility of the information source can be calculated as follows:

$$R_{-}\text{source }=\sqrt{0.6*\left( \frac{1}{20\%} \right)^{2}+0.2*{0.75}^{2}+0.2*\left( \frac{11}{40} \right)^{2}}=3.9 (27)$$

Secondly, calculate the credibility of the information evaluation. When the evaluation of the activity event is none, the evaluation characterizes the credibility of the information as 0; When the evaluation of an activity event is an explicit evaluation, its credibility representation value is directly calculated; When the evaluation of an activity event is calculated as an implicit evaluation, the first step is to determine whether the evaluation is related to the information, and then calculate the tendency value of the relevant representation. The above blockchain calculates the credibility representation values based on evaluation information as follows:

$$R_{-}\text{review }=\sum_{i=1}^{2} \sum\frac{0-0}{2}+\frac{2-1.5}{2}=0.25 (28)$$

The representation of information credibility based on content features is mainly measured by the number of common words, feature words, and professional words in the information content. By conducting relevant statistics on the frequency of various features in enterprise business activity information and calculating them:

$$R_{\text{content }}=\frac{0.2*38+0.3*12+0.5*14}{0.3*64}*\left( \frac{0.2*64}{500} \right)=0.01598 (29)$$

The credibility of information sources is determined by a combination of source based, evaluation based, and content-based credibility. The calculated credibility of information sources is as follows:

$$Accu \left( A \right)=0.5*3.9+0.2*0.25+0.3*0.01598=2.005 (30)$$

Finally, calculate the value information of blockchain:

$$\text{Validity }=-\left( \frac{3}{8}{log}_{2} \frac{3}{8}+\frac{1}{8}{log}_{2} \frac{1}{8}+\frac{1}{2}{log}_{2} \frac{1}{2}+\frac{1}{2}{log}_{2} \frac{1}{2}+\frac{1}{4}{log}_{2} \frac{1}{4}+\frac{1}{8}{log}_{2} \frac{1}{8} \right)=2.782 (31)$$

The final quality evaluation result of blockchain is weighted by three factors:

$$Q=0.1*0.72+0.2*2.005+0.7*2.782=2.4204 (32)$$

The above process can prove that the final quality evaluation model of blockchain in this article has good operability and feasibility.

The experimental dataset consists of two parts, which are official data searched and crawled on the search engine with the subject term of business activities and real data of enterprises, totaling about 110,000 items, and then simple data cleaning is performed by filling in missing values, smoothing or removing outlier points and other operations to get about 100,000 items of data, and the details of the dataset are listed in Table 2. In this paper, simulation experiments are conducted in terms of model parameter taking and model evaluation efficiency respectively, and the model built in this paper (DQAM) is used to compare with other models, and the comparison models are AHP, DSMM, etc.

*Table 2 Experimental data sets*

| *Entity Name* | *Bytes* |
| --- | --- |
| *Name* | *16bytes* |
| *Type* | *8 bytes* |
| *activityOntology* | *Variable length bytes* |
| *comment* | *Variable length bytes* |
| *informationSource* | *16 bytes* |
| *Code* | *4 bytes* |
| *registrationDay* | *4 bytes* |
| *capital* | *16 bytes* |
| *address* | *32 bytes* |
