# Supplementary material for: Multi-source heterogeneous blockchain data quality assessment model for enterprise business activities
Source: PLoS One. 2024 Jun 14;19(6):e0304835. doi: 10.1371/journal.pone.0304835 (PMC11178169; doi:10.1371/journal.pone.0304835)
Supplement: Attachment — Submitted filename: Response to Reviewers-v3.docx [file pone.0304835.s002.docx]

Responses to Reviewers’ Comments

Dear Editor and Reviewers,

Thanks for your comments concerning our manuscript entitled “multi-source heterogeneous blockchain data quality assessment model for enterprise business activities". The comments are very valuable and helpful for revising and improving our manuscript. We have carefully revised the manuscript and responded all comments below. The changes will not affect the content and framework of the paper. The revised sentences are highlighted in yellow in the manuscript.

Best regards,

Baoyan Song

**Reviewer 1:**

**I wanted to inform you that the work you presented is commendable and well-crafted. The previous errors have been addressed effectively, and I find that it has added more clarity and completeness to the research. I look forward to seeing further development in this work.**

**Response：**

First of all, please allow me to express my sincerest gratitude to you. Thank you for taking the time out of your busy schedule to carefully review and evaluate my paper.

Once again, thank you for your attention and support for my paper. Your professional spirit and rigorous attitude have left a deep impression on me, and they also inspire me to continue striving for excellence in my future academic pursuits. I hope to have the opportunity to engage in more academic exchanges with you and jointly promote the development of related fields.

Finally, please continue to follow the progress of my paper. If there are any questions or further modifications needed, please feel free to let me know. I will make the necessary adjustments as soon as possible to ensure the quality and accuracy of the paper.

Thank you once again for your careful guidance and valuable opinions! Wishing you a smooth work and good health!

**Reviewer 3:**

**1. Language is poor, enhance the language and check the miss typos. For example, many words are separated into 2 parts, i.e. (s hortcomings) where “s” separated from “hortcomings”, and so on.**

**Response：**

Thanks for the reviewer 's advice. We have made revisions to the entire text and improved the language expression. The specific modifications are listed below.

*Blockchain-based applications are becoming more and more widespread in business operations.* *In view of the shortcomings of existing enterprise blockchain evaluation methods, this paper proposes a multi-source heterogeneous blockchain data quality evaluation model for enterprise business activities, so as to achieve efficient evaluation of business activity information consistency, credibility and value. This paper proposes a multi-source heterogeneous blockchain data quality assessment method for enterprise business activities, aiming at the problems that most of the data in enterprise business activities come from different data sources, information representation is inconsistent, information ambiguity between the same block chain is serious, and it is difficult to evaluate the consistency, credibility and value of information. The method firstly proposes an entity information representation method based on the Representation learning for fusing entity category information (CEKGRL) model, which introduces the triad structure of related entities in blockchain, then associates them with enterprise business activity categories, and carries out similarity calculation through contextual information to achieve blockchain information consistency assessment. After that, a trustworthiness characterization method is proposed based on information sources, information comments, and information contents, to obtain the trustworthiness assessment of the business. Finally, based on the information trustworthiness characterization, a value assessment method is introduced to assess the total value of business activity information in the blockchain, and a blockchain quality assessment model is constructed. The experimental results show that the proposed model has great advantages over existing methods in assessing inter-block consistency, intra-block activity information trustworthiness and the value of blockchain.*

**2. The introduction needs to be restructured and be more informative to guide the reader, starting with the basic concepts and fundamental definitions and then gradually going through the more related problems and terms. The basic definitions and important clarifications in the introduction should have references. Also, there is redundancy even inside the introduction.**

**Response：**

Thanks for the reviewer 's advice. We have reorganized and revised the contents of the Introduction to make it specifically better logical and readable.

*Blockchain is divided into public chain, private chain, and consortium chain based on its degree of decentralization. Public chain is completely open and transparent, not controlled by any organization, and everyone can participate; The write permission of private chains only belongs to individuals or a certain organization, with a high degree of centralization; The alliance chain, on the other hand, falls between the two and is only open to specific group organizations [8]. Participants can conduct transactions or access information, but only nodes in the alliance have the right to perform transaction verification, publish contracts, and other functions.*

**3. While the research tends more toward the enterprise blockchain data layer rather than traditional) blockchain data layers, it is necessary to includes in the introduction an explanation about this distinction ((with references)).**

**Response：**

Thanks for the reviewer 's advice. We have added a description in the introduction and add a reference 8. The specific modifications are listed below.

*Blockchain is divided into public chain, private chain, and consortium chain based on its degree of decentralization. Public chain is completely open and transparent, not controlled by any organization, and everyone can participate; The write permission of private chains only belongs to individuals or a certain organization, with a high degree of centralization; The alliance chain, on the other hand, falls between the two and is only open to specific group organizations [8]. Participants can conduct transactions or access information, but only nodes in the alliance have the right to perform transaction verification, publish contracts, and other functions.*

**4. Many sentences are long and take place over three or more lines. It is preferable to shorten the length of sentences, for example, by breaking them into 2 or more sentences.**

**Response：**

Thanks for the reviewer 's advice. We have revised the full text description by breaking down long sentences into short ones, making the paper more readable. The specific modifications are listed below.

*Moreover, traditional evaluation methods do not make full use of the features of blockchain. Blockchain features include leaving traces throughout the process, being non-temper able, and traceable. Evaluation efficiency and accuracy are low with traditional methods. This results in the inability of enterprise users and relevant regulatory authorities to quickly screen out suitable blockchains. Establishing a unified analysis model becomes challenging [9].*

**5. The author, in introduction wrote “Large domestic and foreign enterprise units ..”, such sentence is not clear!, at first which of the mentioned example are domestic ? and domestic relating for whom?, and what is the meaning of “units” in such contexts?.**

**Response：**

Thanks for the reviewer 's advice. We change the description in the introduction. The specific modifications are listed below.

*Many IT companies around the world, such as IBM, Baidu and Alibaba, have established their own enterprise federated blockchain systems.*

**6. In the introduction the author mention that “ … Google, Baidu and Alibaba, have established their own enterprise federated blockchain systems”. But they do not provide reference for such examples! For instance, it is well known that Google's offers Blockchain-as-a-Service (BaaS) which is primarily support public blockchains, but the Authors need to provide references supports their examples about federated blockchains.**

**Response：**

Thanks for the reviewer 's advice. We have changed the description. The specific modifications are listed below.

*Many IT companies around the world, such as IBM, Baidu and Alibaba, have established their own enterprise federated blockchain systems.*

**7. As a continuation of the previous note, the authors already mention (federated blockchains) without explanation, and why it is related to their work. So, the authors also prefer to insert brief definitions of Public, private, and Federated Blockchains in the introduction, making clear the differences between them.**

**Response：**

Thanks for the reviewer 's advice. We have added a description in the introduction. The specific description is listed below.

*Blockchain is divided into public chain, private chain, and consortium chain based on its degree of decentralization. Public chain is completely open and transparent, not controlled by any organization, and everyone can participate; The write permission of private chains only belongs to individuals or a certain organization, with a high degree of centralization; The alliance chain, on the other hand, falls between the two and is only open to specific group organizations [8]. Participants can conduct transactions or access information, but only nodes in the alliance have the right to perform transaction verification, publish contracts, and other functions.*

**8. In the related works, the author listed some references under works that is specifically (In blockchain information consistency evaluation), but it not!!! For example, (and not limited to), the reference number (14) is related for natural language processing and the author of (14) dose not mentions any explicitly relationship with Blockchain!!!. Without a clear connection or application area identified within the blockchain, the direct applicability is limited. ((((The authors should reanalyze all the related works again and focus on the similarities within the area of challenges to determine the feasibility of such an application)))). The author should recheck all the related works!. All of them should have direct relationship with the proposed work.**

**Response：**

Thanks for the reviewer 's advice. We have reanalyzed all the related works again. The specific modifications are listed below.

*This paper introduces a novel approach: a multi-source, heterogeneous blockchain quality assessment model tailored for enterprise business activities. It addresses the limitations of current methods by prioritizing consistency, credibility, and data value [22]. This model enhances blockchain consistency assessment efficiency while also factoring in content credibility, yielding promising results even in scenarios with high information uncertainty.*

**9. I could not find some cited papers in any resource in the internet !!! for example (Disambiguation method of inconsistent records based on factor graph)!!!???? Please provide the correct link, and explain this. I miss something here?!!!!.**

**Response：**

Thanks for the reviewer 's advice. We have corrected the incorrectly cited papers. The specific modifications are listed below.

*[8] Zheng Peilin, Xu Quangqing, ZHENG Zibin, et al. (2021) Meepo: Sharded consortium blockchain. 2021 IEEE 37th International Conference on Data Engineering, Chania, Greece, 1847–1852*

*[12] Yi Yang, Ozan Irsoy, and Kazi Shefaet Rahman. (2018) Collective Entity Disambiguation with Structured Gradient Tree Boosting. In Proceedings of the 2018 Conference of the North American Chapter of the Association for Computational Linguistics: Human Language Technologies, Volume 1, New Orleans, Louisiana. Association for Computational Linguistics, 777–786.*

*[14] Jiang Jing, Wang Kai, Xu Yueqiang, et al. (2022) Optimal Caching Strategy of Operators Based on Consortium Blockchain [J]. Journal of Electronics & Information Technology, 44 (9): 3043-3050.*

**10. In the related works, the authors cited the preprint version of a paper (the reference number 12) while this work have official published version:** <https://aclanthology.org/N18-1071/> **. The authors need to focuses on the officially published version and analysis it again.**

**Response：**

Thanks for the reviewer 's advice. We have reanalyzed and cited reference 12.

*[12] Yi Yang, Ozan Irsoy, and Kazi Shefaet Rahman. (2018) Collective Entity Disambiguation with Structured Gradient Tree Boosting. In Proceedings of the 2018 Conference of the North American Chapter of the Association for Computational Linguistics: Human Language Technologies, Volume 1, New Orleans, Louisiana. Association for Computational Linguistics, 777–786.*

**11. Any fundamental used terms, for example (and not limited to) the triple structure, better to be well defined in the introduction or in the related works with citations.**

**Response：**

Thanks for the reviewer 's advice. The concept of triple structure is proposed in this article. In order to distinguish from the existing definition, this article is modified to "Triple Evaluation Structure".

*The triple evaluation structure is a data model widely used for representing information, consisting of three components: subject, predicate, and object. It is extensively applied in fields such as knowledge graphs and the semantic web. The triple evaluation structure is highly flexible and can be employed to represent various types of information. Different combinations of subjects, predicates, and objects can capture rich semantic relationships, making it suitable for diverse knowledge representation. In summary, the advantages of the triple evaluation structure lie in its flexibility, clear semantic representation, scalability, and effective expression of relationships, making it a key technology in the fields of knowledge representation and the semantic web.*

**12. The third section needs to reconsider the structure of its sections. The logical connection between its sub-sections must be clearer. Perhaps there is a need for a workflow in the form of an illustration (new figure) or something similar.**

**Response：**

Thanks for the reviewer 's advice. We add a logic flowchart to show our technical roadmap.

*Fig 1. The Logic flowchart of Showcase Assessment Model technical roadmap*

**13. Let sub-section 3.7 have more informative title.**

**Response：**

Thanks for the reviewer 's advice. We change the title as “Case analysis of enterprise alliance chain evaluation”.

**14. It is necessary to provide an explanation of each data set used in the experiment, along with a reference or link to these data sets.**

**Response：**

Thanks for the reviewer 's advice. We have uploaded the dataset of this paper to the figshare database and added the generated doi to the content of the experimental setup. The specific modifications are as follows.

*The experimental dataset consists of two parts, which are official data searched and crawled on the search engine with the subject term of business activities and real data of enterprises, totaling about 210,000 items, and then simple data cleaning is performed by filling in missing values, smoothing or removing outlier points and other operations to get about 200,000 items of data, and the details of the dataset are listed in Table 2. The datasets are available from https://doi.org/10.6084/m9.figshare.25143503.v1.*

**15. It is important to provide a clear explanation regarding the comparison with reference to the related works, include citations.**

**Response：**

Thanks for the reviewer 's advice. We have improved the literature and analysis of the relevant work, supplemented the latest technologies and methods, analyzed the advantages and disadvantages, added citations throughout the text, and compared them with the methods proposed in this paper in experiments.

*This section compares the running efficiency of each model, the horizontal coordinate represents the entity data set in the blockchain, in units of ten thousand, the vertical coordinate indicates the time required to run each model, and the experimental results are shown in Fig 8.*

**16. In fact, I cannot agree with what is stated in the conclusion, because there is a major weakness in the study of related works.**

**Response：**

Thanks for the reviewer 's advice. We have improved the literature and analysis of the relevant work, supplemented the latest technologies and methods, analyzed the advantages and disadvantages, and compared them with the methods proposed in this paper in experiments.

*Initially, leveraging pertinent enterprise entity data within the blockchain, we introduce a triadic representation of enterprise entities utilizing the CEKGRL model, intricately linked with relevant activity categories. This integration enables the construction of an association graph model for entity context information, thus enhancing the efficiency of similarity computations between blocks. Subsequently, through an examination of blockchain content, we ascertain the credibility of blockchain information via a credibility characterization method. This method is grounded in information source analysis, information commentary evaluation, and the content of business activity information, amalgamating all characterization outcomes to provide a comprehensive credibility assessment.*

**Reviewer 4:**

**1. The second contribution indicated in the introduction should be looked at and rewritten well. it's confusing which basis that statement is based on.**

**Response：**

Thanks for the reviewer 's advice. We have made revisions and improvements to the second contribution. The specific modifications are as follows.

*In response to the challenge of assessing the credibility of business information on the blockchain, we introduce a novel method for characterizing information trustworthiness that integrates the source, evaluative features, and content features. This method not only verifies the credibility of the information source through an assessment of the trustworthiness of the publishing platform, page, and publisher but also generates a comment-based credibility representation by analyzing comment tendencies based on lexicon types. Furthermore, it achieves a content-based credibility representation by examining the content characteristics of business activities. This holistic approach provides a comprehensive solution for information evaluation within the blockchain ecosystem.*

**2. The summary of the introduction should be rewritten because it is too long and makes it difficult to get what you are communicating. some more sentences are long making reading them difficult, and they should be looked at.**

**Response：**

Thanks for the reviewer 's advice. We have revised the full text description by breaking down long sentences into short ones, making the paper more readable. The specific modifications are as follows.

*This paper introduces a novel approach: a multi-source, heterogeneous blockchain quality assessment model tailored for enterprise business activities. It addresses the limitations of current methods by prioritizing consistency, credibility, and data value [22]. This model enhances blockchain consistency assessment efficiency while also factoring in content credibility, yielding promising results even in scenarios with high in-formation uncertainty.*

**3. The literature survey must be comprehensive. Add more existing literature.**

**Response：**

Thanks for the reviewer 's advice. We have improved the literature and analysis of the relevant work, supplemented the latest technologies and methods, analyzed the advantages and disadvantages, added citations throughout the text, and compared them with the methods proposed in this paper in experiments. The specific references added are listed below.

*[8] Zheng Peilin, Xu Quangqing, ZHENG Zibin, et al. (2021) Meepo: Sharded consortium blockchain. 2021 IEEE 37th International Conference on Data Engineering, Chania, Greece, 1847–1852*

*[12] Yi Yang, Ozan Irsoy, and Kazi Shefaet Rahman. (2018) Collective Entity Disambiguation with Structured Gradient Tree Boosting. In Proceedings of the 2018 Conference of the North American Chapter of the Association for Computational Linguistics: Human Language Technologies, Volume 1, New Orleans, Louisiana. Association for Computational Linguistics, 777–786.*

*[14] Jiang Jing, Wang Kai, Xu Yueqiang, et al. (2022) Optimal Caching Strategy of Operators Based on Consortium Blockchain [J]. Journal of Electronics & Information Technology, 44 (9): 3043-3050.*

**4. it will be appreciated if the experimental dataset is made available on a publicly accessible repository with a correct or accessible doi. the one provided here cannot be assessed.**

**Response：**

Thanks for the reviewer 's advice. We have uploaded the dataset of this paper to the figshare database and added the generated doi to the content of the experimental setup. The specific modifications are as follows.

*The experimental dataset consists of two parts, which are official data searched and crawled on the search engine with the subject term of business activities and real data of enterprises, totaling about 210,000 items, and then simple data cleaning is performed by filling in missing values, smoothing or removing outlier points and other operations to get about 200,000 items of data, and the details of the dataset are listed in Table 2. The datasets are available from https://doi.org/10.6084/m9.figshare.25143503.v1.*

**5. In the references section, all references must be of the same style and must be complete eg. Check reference # 21**

**Response：**

Thanks for the reviewer 's advice. We have checked the style of all references and modified it to a uniform style. The specific modifications are as follows.

*[1] He X, Wang J, Liu J, Yuan E, Wang K, Han Z (2021) Smart Grid Nontechnical Loss Detection Based on Power Gateway Consortium Blockchain.*

*[2] Shen M, Sang A Q, Zhu L, Sun R, Zhang C (2021) Recognition method of Abnormal Transaction Behavior of Blockchain digital Currency based on Motivation Analysis. Journal of Computers, 44(01): 193-208.*

*[3] Hong S (2021) Research on Sharding Model for Enabling Cross Heterogeneous Blockchain Transactions. Journal of Digital Con-vergence 19(5): 315-320.*

*[4] Fu L, Tian H (2019) Ethereum Coin Voting Protocol Based on Smart Contract. Journal of software, 30(11): 3486-3502.*

*[5] Wang X, Yang X, Shu X, Zhao L (2021) Formal verification of smart Contract for MSVL. Journal of software, 32(6): 1849-1866.*

*[6] Truong N, Lee GM, Sun K, Guitton F, Guo Y (2021) A blockchain-based trust system for decentralized applications: When trust-less needs trust. Future Generation Computer Systems, 124: 68-79.*

*[7] Lee G M (2021) A blockchain-based trust system for decentralized applications: When trustless needs trust. Future Generation Computer Systems: the international journal of grid computing: theory, methods and applications 124: 68-79.*

*[8] Zheng Peilin, Xu Quangqing, ZHENG Zibin, et al. (2021) Meepo: Sharded consortium blockchain. 2021 IEEE 37th International Conference on Data Engineering, Chania, Greece, 1847–1852.*

*[9] Colomo‐Palacios R, Sánchez‐Gordón M, Arias‐Aranda D (2020) A critical review on blockchain assessment initiatives: A technology evolution viewpoint. Journal of Software: Evolution and Process 32(11): e2272.*

*[10] Zhang A, Zhong RY, Farooque M, Kang K, Venkatesh VG (2020) Blockchain-based life cycle assessment: An implementation framework and system architecture. Resources, Conservation and Recycling 152: 104512.*

*[11] Zheng Z, Shao S, Gao X, Chen G (2021) Prediction of information Heat based on social circle and attention mechanism. Journal of Computers 44(05): 921-936.*

*[12] Yi Yang, Ozan Irsoy, and Kazi Shefaet Rahman. (2018) Collective Entity Disambiguation with Structured Gradient Tree Boosting. In Proceedings of the 2018 Conference of the North American Chapter of the Association for Computational Linguistics: Human Language Technologies, Volume 1, New Orleans, Louisiana. Association for Computational Linguistics, 777–786.*

*[13] Yang Y, Irsoy O, Rahman K S (2018) Collective entity disambiguation with structured gradient tree boosting. arXiv preprint arXiv:1802.*

*[14] Jiang Jing, Wang Kai, Xu Yueqiang, et al. (2022) Optimal Caching Strategy of Operators Based on Consortium Blockchain [J]. Journal of Electronics & Information Technology, 44 (9): 3043-3050.*

*[15] Wang C, Sun X, Yu H, Zhang W (2019) Entity disambiguation leveraging multi-perspective attention. IEEE Access 7: 113963-113974.*

*[16] Huo X, Zhang Y, Jing Y, Shao K (2020) A Multi-dimensional credibility Calculation Method based on feedback credibility in MAS Environment. Journal of software 31(02): 374-394.*

*[17] Feng Y, Hu J, Duan R, Chen Z (2021) Credibility Assessment Method of Sensor Data Based on Multi-Source Heterogeneous In-formation Fusion. Sensors 21(7): 2542-2542.*

*[18] Jain P K, Pamula R, Ansari S. A (2021) Supervised Machine Learning Approach for the Credibility Assessment of User-Generated Content. Wireless Personal Communications 118(4): 2469-2485.*

*[19] Moon MY, Cho H, Choi KK, Gaul N, Lamb, D, Gorsich D (2018) et al. Confidence-based reliability assessment considering limited numbers of both input and output test data. Structural and Multidisciplinary Optimization 57(5): 2027-2043.*

*[20] Lin C, Zhang M, Zhou Z, Li L, Shi S, Chen Y, Dai W (2020) A new quantitative method for risk assessment of water inrush in karst tunnels based on variable weight function and improved cloud model. Tunnelling and Underground Space Technology 95: 103136.*

*[21] Xiao L, Huang G, Zhang G (2021) Improved assessment model for candidate design schemes with an interval rough integrated cloud model under uncertain group environment. Engineering Applications of Artificial Intelligence 104: 104352.*

*[22] Wang J, Zhang R, Ji W et al (2022). Research on quality assessment method of blockchain multi-source heterogeneous business data.*
